# Potential of Lipid-Based Nanocarriers against Two Major Barriers to Drug Delivery—Skin and Blood–Brain Barrier

**DOI:** 10.3390/membranes13030343

**Published:** 2023-03-16

**Authors:** Mohammad Sameer Khan, Sradhanjali Mohapatra, Vaibhav Gupta, Ahsan Ali, Punnoth Poonkuzhi Naseef, Mohamed Saheer Kurunian, Abdulkhaliq Ali F. Alshadidi, Md Shamsher Alam, Mohd. Aamir Mirza, Zeenat Iqbal

**Affiliations:** 1 School of Pharmaceutical Education and Research (SPER), Jamia Hamdard, New Delhi 110062, India; 2 Nanotechnology Lab, School of Pharmaceutical Education and Research (SPER), Jamia Hamdard, New Delhi 110062, India; 3 Department of Pharmaceutics, Moulana College of Pharmacy, Perinthalmanna, Kerala 679321, India; 4 Department of Dental Technology, College of Applied Medical Sciences, King Khalid University, Abha 61421, Saudi Arabia; 5 Department of Pharmaceutical Chemistry, College of Pharmacy, Jazan University, Jazan P.O. Box 114, Saudi Arabia

**Keywords:** biological barriers, drug delivery, lipid-based nanocarriers, BBB, skin, solid lipid nanoparticles (SLN), nanostructured lipid carriers (NLC)

## Abstract

Over the past few years, pharmaceutical and biomedical areas have made the most astounding accomplishments in the field of medicine, diagnostics and drug delivery. Nanotechnology-based tools have played a major role in this. The implementation of this multifaceted nanotechnology concept encourages the advancement of innovative strategies and materials for improving patient compliance. The plausible usage of nanotechnology in drug delivery prompts an extension of lipid-based nanocarriers with a special reference to barriers such as the skin and blood–brain barrier (BBB) that have been discussed in the given manuscript. The limited permeability of these two intriguing biological barriers restricts the penetration of active moieties through the skin and brain, resulting in futile outcomes in several related ailments. Lipid-based nanocarriers provide a possible solution to this problem by facilitating the penetration of drugs across these obstacles, which leads to improvements in their effectiveness. A special emphasis in this review is placed on the composition, mechanism of penetration and recent applications of these carriers. It also includes recent research and the latest findings in the form of patents and clinical trials in this field. The presented data demonstrate the capability of these carriers as potential drug delivery systems across the skin (referred to as topical, dermal and transdermal delivery) as well as to the brain, which can be exploited further for the development of safe and efficacious products.

## 1. Introduction

In the human body, biological barriers such as the skin, blood–brain barrier (BBB), mucosal membranes and membranes around cells and nuclei etc, limit the delivery of drugs to the desired sites to achieve the necessary therapeutic actions. On the one hand, these barriers protect the human body from invading pathogens and on the other hand, they limit drug transport. Therefore, much research has been explored to overcome this obstacle which in general involves two main approaches, including the formulation-based and device-based approaches. In the device-based approach, novel devices have been designed, often operated by expensive power sources, to overcome the barriers and impediments. They disrupt the structure of the organ temporarily and allow for the delivery of macromolecules through it [1,2,3]. Although this technique is successful but expensive, it often needs a power supply for its operation, is risky and is not patient compliant. Further, it is limited to selective accessible organs [4]. However, the formulation-based approach (non-invasive method) involves designing newer formulations involving pro-drugs, new excipients, permeation enhancers or constructing novel delivery systems to transport the drugs across these barriers [4,5]. This review discusses the formulation-based approach involving lipid-based nanocarriers specifically for two major biological barriers such as the skin and the BBB.

Skin (the largest organ of the body) is comprised of three distinct layers including the epidermis (outermost layer); dermis (middle layer); and hypodermis (innermost layer). The epidermis is further divided into different layers and is primarily composed of keratin, which acts as a substantial barrier to drug permeation and has a thickness of 53 μm [6]. The superficial layer of the epidermis consists of a stratum corneum, which is a physically and compositionally unique structure. This thin, least permeable skin layer is formed in the last stage of the epidermal differentiation process, making a laminate of compressed keratin-filled corneocytes attached to a lipophilic matrix [7,8]. The overlapped layout of corneocytes in the lipid layer is suggested to be responsible for the highly distorted lipoidal diffusion path, making it comparatively less permeable to water than other biomembranes [9] and is believed to be the major obstacle for drug transportation. It acts as a permeation barrier for the molecules that are of a size greater than 500 Da [10]. Other factors such as low pH, the transcutaneous concentration gradient and the presence of enzymes on the skin are the additional attributes of the epidermal stratum corneum that negatively influence the skin penetration of drugs [11,12]. Further, the size, shape, lipophilicity, surface charges, presence of penetration enhancers, physical state of the stratum corneum and type of formulation are the additional considerations that may affect the absorption of topically applied agents through the skin [13,14]. Apart from these, there are other factors that can affect drug delivery including the physicochemical characteristics of the permeant (drug); the physicochemical characteristic of the vehicle in which the permeant (drug) is dispersed; and the dosing conditions [15]. Because of these shortcomings in the traditional routes of drug administration, significant strategies have been made which can be enlisted as a passive and active enhancement to circumvent the barrier property of the stratum corneum [15] accompanied by nanotechnology. Currently, the topical administration of the drugs through various layers of skin and appendages comes to be an appealing area of research as it provides several unique delivery advantages [16].

The delivery of drugs to the central nervous system (CNS) has been a challenging as well an active area of drug development, attributed to the presence of the BBB [17]. This is basically an extremely selective structural and biochemical barrier that preserves the intracerebral environment supplying blood (nutrients) to the brain and protecting against the environment [18]. It plays a crucial role in protecting the brain parenchyma and provides a significant hindrance to the entry of exogenous compounds into the CNS [19]. The presence of the BBB protects the brain, maintains neuronal activity and protects the brain’s homeostasis, but simultaneously poses a challenge for drug distribution across this barrier. Many traditional neurotherapeutic agents are unable to cross the BBB in appreciable concentrations, thus limiting the brain’s uptake [20]. There are several strategies such as chemical modifications of the drugs (for improving their lipophilicity), use of molecular tools to bind the drugs to small immunoglobulins, and nanotechnology using vesicles, liposomes and micelles are employed to deliver drugs to the brain. More research has been conducted to develop novel therapeutics for the effective treatment of diseases affecting the CNS [21]. Recently, lipid-based nanocarriers have been used in key strategies for CNS drug delivery. These types of delivery systems improve the permeability of active agents with enhanced half-lives and stability by maintaining the required drug levels in the systemic circulation. Additionally, these systems can either trigger or control the rate of release of therapeutic ingredients in the blood by attaining the requirement of the patients.

The burgeoning application of lipid-based nanocarriers in pharmaceutical formulations for the delivery of diversified therapeutic agents is gaining momentum over the last decades [22]. They have the capability of permeating the therapeutic loads through the skin as well as the BBB owing to their lipophilic nature. These nanocarriers significantly augment the solubility (lipophilic as well as hydrophilic agents) and permeability, and hence the bioavailability of the loaded agents. Nanocarriers, owing to their small particle size, high surface–volume ratio, unique physicochemical properties and capability to cross different biological barriers, are gaining considerable interest in pharmaceutical research [23,24]. Additionally, they show several advantages such as easy fabrication without using organic solvents, storage stability, the capability of lyophilization and steam sterilization, biocompatibility and biodegradability, etc. Some of the advantages of these novel lipid-based nanocarrier systems can also be extended to the skin and brain delivery of drugs for achieving local (dermal), systemic (transdermal) and targeted effects [25].

This review includes a discussion of several lipid-based nanocarriers, their composition and applications. It also includes recent research and development findings (including recent patents and clinical trials) regarding the use of lipid-based drug delivery systems across the skin and BBB for disease treatment. This work also summarizes recent findings from different known databases, which are Google Scholar, Google patents, Science Direct, MEDLINE (PubMed), Scopus and SciFinder. This article compiles the latest progress in the performances and advantages of the said carrier systems in dealing with these interesting yet challenging biological barriers. This not only generates evidence to support the applications of these delivery systems against the major biological barriers of the human body, but also encourages future research in this arena for more successful therapeutic outcomes by overcoming these challenges.

## 2. Lipid-Based Nanocarrier Systems

Rampant applications of multifunctional nanoparticles in the pharmaceutical arena have gained attention in the last few decades, which have led to the accelerated development of therapeutic and imaging agents [26]. For the successful delivery of a drug, these nano-level carriers play an important role by incorporating active moieties with them. They enable the molecules to reach the desired site either in a sustained or controlled way depending on the requirement of the therapy. The nanocarriers allow for the delivery of the drugs with improved efficiency of the existing as well as new drugs with minimal obstacles [27]. Amongst them, lipid-based nanocarriers are widely used nowadays, owing to their unique advantages compared to the other traditional carrier systems.

Solid lipids are the main components of lipid-based nanocarriers. Depending on the chemical composition of the lipids they can be classified as homolipids, heterolipids and complex lipids. Homolipids are known as simple lipids containing esters of fatty acids and alcohol. They contain only carbon (C), hydrogen (H) and oxygen (O), and are the primary materials of interest for oral delivery vehicles. The long-chain fatty acids (ranging from C14 to C24) appear usually in common fat while the medium-chain fatty acids (ranging from C6 to C12) are typical elements of coconut oil or palm kernel oil. Beeswax, carnauba wax, glycerides and sterides are examples of homolipids. Heterolipids, also known as compound lipids, are lipids containing nitrogen (N) and phosphorus (P) atoms in addition to the C, H and O. Phospholipids that are widely used in pharmaceuticals are an example of this type of lipid. Two main classes of phospholipids that occur naturally in qualities sufficient for pharmaceutical applications are phosphoglycerides and phosphosphingolipids. Some phosphosphingolipids such as ceramide are used mainly in topical dosage forms. Phospholipids can be obtained from all types of biomasses as they are essential structural components in all kinds of membranes in living organisms. Glycolipids and sulfolipids are other two examples of heterolipids. The complex lipids occur closely associated with proteins present in cell membranes and subcellular particles. Active tissues generally have a higher complex lipid content and may also contain phospholipids, lipoproteins, chylomicrons, etc. Lipoproteins are spherical lipid–protein complexes that are responsible for the transport of cholesterol and other lipids within the body [28].

Table S1 depicts specific lipid-based carriers that enable crossing of the skin and brain barriers. These illustrations generate evidence to support the efficiency of these carriers in overcoming the barrier functions of the skin and brain and support their future applications.

**Figure 1:**
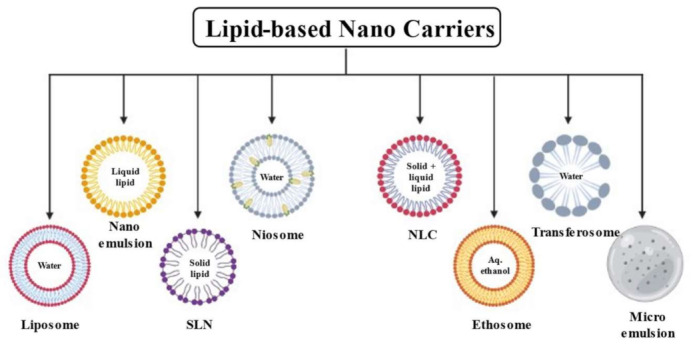
List of lipid-based nanocarriers used for delivery of pharmaceutically active agents.

Lipids are hydrophobic molecules which are insoluble in water and have been used for making different pharmaceutical formulations. They improve the solubility, absorption, permeation and hence the bioavailability of poorly soluble drugs with relatively less-toxic effects. The biocompatibility, biodegradability and nontoxic nature of these molecules make them more popular for the designing of nanocarriers for biomedical uses [29]. Different types of lipid-based nanocarrier systems such as liposomes, solid lipid nanoparticles, nanostructured lipid carriers, lipid–polymer hybrid nanoparticles and phytosomes can be produced depending on the content of lipids/excipients and the formulation technique employed [30]. Figure 1 depicts different available lipid carriers for the delivery of pharmaceutically active agents.

Although the exact mechanism of action for the penetration of these systems is not known, some of the mechanisms of such vesicles in altering the skin permeability have been suggested, such as (i) penetration of the whole drug-loaded vesicle into the layers of the skin; (ii) they might act as penetration enhancers by fluidizing skin lipids; (iii) direct carrier–skin drug exchange by “collision complex transfer”; and (iv) lipid vesicle-interceded drug delivery through skin appendages such as hair follicles and sweat ducts. Besides all these functions, they enhanced the adhesiveness, hydration, occlusion and lubrication of the skin [31,32,33,34,35]. Adhesiveness is a basic desirable property for the hydration of the skin. This increases the time of contact of the drug over the skin and permits cutaneous absorption. The smaller the size the greater the surface area for skin adhesion. Hence, lipid-based nanocarriers are more effective than conventional topical formulations by causing both occlusion and hydration on the skin surface [36,37,38].

Further, the smaller size of these nanocarriers (10–200 nm) allows them to cross the BBB and penetrate through the brain by the endocytosis mechanism [39]. Additionally, passive targeting and active targeting of the CNS are based on non-altered surface nanoparticles and the functionalization of the surface with targeting ligands (maintaining the concentration gradient across the BBB), which respectively help to penetrate the drug through the brain [40,41]. Other than endocytosis, transcytosis and receptor-mediated transport are the other mechanisms by which drug molecules enter the brain.

Lipid-based nanocarrier systems are highly biocompatible and biodegradable systems, successfully used as carriers for poorly water-soluble drugs and oligonucleotides (DNA/RNA) in gene therapy. Further, they can be used for encapsulating active ingredients for targeted drug delivery in the body. Table 1 conscripts a list of marketed formulations incorporating lipid carriers for several disorders through various routes. This generates proof for the wide applications of lipid carriers for managing different ailments in humans and encourages the formulator to conduct more investigation in this area.

The following section describes different lipid nanocarriers used in the pharmaceutical arena.

### 2.1. Liposome

Liposomes are small, spherical vesicles that contain natural or synthetic non-toxic phospholipids as their key ingredients. They have an aqueous core surrounded by a hydrophobic lipid bilayer membrane in such a way that the hydrophilic solutes dissolved in the core cannot readily pass through the bilayer. Both hydrophilic and hydrophobic molecules can be loaded in liposomal vesicles and hence can be used for drug delivery. The liposomes can be classified as unilamellar vesicles (having one bilayer membrane), oligo lamellar vesicles (having 2–5 bilayer membranes) and multilamellar vesicles (having 5 or more bilayer membranes). Unilamellar vesicles are further subdivided into three categories that include small unilamellar vesicles, large unilamellar vesicles and giant lamellar vesicles. Among these, unilamellar vesicles are mostly used in drug delivery because of their small size (e.g., nanosized range), uniform drug capsulation and they release kinetics together with long circulation times [57]. The distribution of these molecules to the site of action occurs by the fusion of lipid bilayers with the cell membrane, thus delivering the liposomal contents [57]. Liposomes can be fabricated by the thin-film method, detergent removal method, pro-liposome method, solvent injections method, reverse phase evaporation method and emulsification method, etc. [58]. Occasionally, post-formation processing is employed to further reduce the size of the vesicles to achieve specific objectives. Sonication, high-pressure homogenization and extrusion are the most widely used methods that are used for post-formation downsizing of the vesicles in this liposome formulation approach [59]. Currently, novel technologies such as lyophilization, supercritical fluid-assisted technology, microfluidic, membrane contactor methods, etc. are employed to avoid the critical issues related to traditional methods [59]. The advantages of liposomes include their biocompatibility, biodegradability, drug delivery specifically to sensitive tissues, their flexibility to couple with site-specific ligands for achieving active targeting and their improved stability owing to encapsulation, etc. However, their low solubility, short half-life, high production cost, poor stability, etc. are a few of the disadvantages associated with them [60].

The majority of the conventional liposomes are restricted to the first layer of the epidermis and are unable to reach systemic circulation. Hence, they are restricted to use in local dermal drug delivery and demand new classes of nano lipid vesicles for enhancing flexibility and skin permeation. However, in the case of the brain, the interstitium is dense and highly charged, thus restricting the movement of many classes of nanocarriers within the CNS. This issue can be circumvented by liposomes with surface modifications that mask the particle charge, thus allowing for more uniform brain penetration. Secondly, the modification of nanocarriers with targeting moiety can enhance retention in a diseased site. Therefore, the disrupted BBB can be targeted with liposomes that are specifically formulated for penetration and retention at the diseased location [61].

Liposomal formulations can be further categorized into (1) rigid vesicles (liposomes and niosomes) and (2) elastic or ultra-deformable vesicles (transferosomes and ethosomes). Their composition, structure and preparation methods are more or less similar; however, their differences relate to their deformability and mechanism of penetration through the skin [62].

### 2.2. Niosomes

These are non-ionic surfactant vesicles composed of single-chain surfactant molecules in combination with cholesterol. These surfactants form an enclosed bilayer in a water medium. The presence of surfactant alters the permeation through biological barriers and makes it more penetrable with enhanced systemic absorption. These systems are capable of carrying both lipophilic and hydrophilic drugs and comparatively more stable than traditional liposomes [63]. They offer better drug concentrations at the site of action administered by several routes such as parenteral, oral and topical. Drugs with a low therapeutic index and low water solubility can be utilized for sustained action through these delivery systems, but simultaneously they have problems like clustering, fusion and leaking. Sonication, micro fluidization, thin-film hydration, reverse phase evaporation, ether injection, trans-membrane pH gradient drug uptake (remote loading) and multiple membrane extrusion are various methods used for preparing these vesicles [64,65,66].

Niosomes can penetrate into the skin by adsorption onto the cell surface with little or no internalization of either aqueous or lipid components. It may take place either as a result of attracting physical forces or as a result of binding by specific receptors to ligands on the vesicle membrane, thus transferring the drug directly from vesicles to the skin. Further, niosomes may fuse with the cell membrane, resulting in the complete mixing of the niosomal contents with the cytoplasm. Lastly, niosomes may undergo endocytosis, with lysozymes present in the cytoplasm degrading the membranous structure of the niosome, thereby releasing the entrapped material into the medium vehicle, which can be achieved by forming a niosomal gel [67]. Niosomes have also the capability to overcome the BBB and access drug delivery to the brain by surface modification. They improve the therapeutic performance of the drug by surface modification and limiting the effects to target cells, thus decreasing the clearance of the drug [68].

### 2.3. Ethosome

These are non-invasive, phospholipid-containing nano lipid carriers that are more efficient at delivering drug molecules into the skin in terms of both the quantity and depth as compared to the other vesicles. They are mainly composed of lipids, ethanol and water. The percentage of ethanol in this vesicular system has been reported to be in the range of 10–50% [5,69]. It has been investigated that with an increase in ethanol concentration in this range, the vesicular size decreases [70,71]. These vesicle systems differ from liposomes as they include high concentrations of ethanol [72]. The mechanism of penetration of the ethosomes involves two concurrent mechanisms, which include the effect of ethanol and ethosomal vesicles on the stratum corneum lipid bilayer that will enhance the delivery of molecules through the skin [73].

These can be prepared by four principal methods including the cold method; hot method; classic method; and mechanical dispersion method [73,74]. These are relatively safe, biocompatible and friendly carriers used to deliver large molecules through the skin with an enhanced permeation and good therapeutic index. They have a large market share in the pharmaceutical, veterinary and cosmetic fields. On the other hand, they are limited only to potent molecules that require high blood levels; they are not a means to achieve rapid bolus-type drug input, rather they are usually designed to offer slow, sustained drug delivery; drugs having an adequate solubility in both lipid and aqueous environments are capable of reaching dermal microcirculation and gain access to the systemic circulation [73,75].

The enhanced delivery of drugs through ethosomes may be ascribed to an interaction between ethosomes and skin lipids. The presence of ethanol interacts with the polar heads of the lipid molecules, resulting in a reduction in the transition temperature of the lipids present in the stratum corneum, thus improving their fluidity and reducing the density of the lipid layers [75,76,77]. This is followed by the fusion of ethosomes to the skin lipids which results in their penetration and permeation by the opening of new pathways. This is mainly due to the malleability and fusion of ethosomes on the skin lipids which results in the permeation of the drug into the deep layers of the skin. The presence of ethanol makes the vesicles soft and flexible, which allows them to penetrate more easily into the deeper layers of the skin and results in systemic absorption [78].

### 2.4. Transethosome

Transethosomes are a combination of transferosomes and ethosomes. Transferosomes are composed of phospholipids and an edge activator (single-chain surfactant) that weakens the lipid bilayers and increases their deformability by reducing the interfacial tension. These have a deformable quality as well as a skin permeation capability and can be taken up by systemic as well as topical routes. Ethanol is a central character of transethosomal systems, giving a unique identity to them as a vesicular system. It is believed that the first part of the mechanism is due to the “ethanol effect”, whereby the intercalation of the ethanol into intercellular lipids increases lipid fluidity and decreases the density of the lipid bilayer. The lipid layer of the stratum corneum is fluidized by the impact of ethanol and its high concentration in transethosomes promotes the malleability and flexibility of these systems, enhancing their penetration through tiny openings formed in the stratum corneum due to fluidization. The presence of the alcohol amount in the vesicular system also controls its diameter, as it provides a net negative charge to the vesicle surface by reducing its size [79]. Transethosomes can be prepared by various methods such as ethanol injection, the thin-layer hydration method, cold, direct and reverse-phase evaporation methods [80]. Transethosomes are stable, non-invasive vesicles having a high patient compliance as they can be administered in semisolid dosage forms such as gels, creams, lotions, etc. On the other hand, they cause occasional skin irritation and other allergic reactions as ethanol is used in their formulation.

### 2.5. Solid Lipid Nanoparticles (SLNs)

SLNs are one of the novel carriers in the pharmaceutical industry for the delivery of multiple drugs [81,82]. SLNs are generally spherical in shape, whose size ranges from 100 nm to 1000 nm. The central portion of the SLNs is made up of lipid (solid-state) which is well stabilized by the emulsifiers encapsulating the lipidic molecules in a stable lipid matrix [83]. SLNs can be prepared with different techniques such as high shear homogenization and ultrasound, solvent emulsification, solvent evaporation and microemulsion [84]. The advantages of SLNs include improved skin penetration, prolonged release of the drug from formulation that takes place owing to erosion of the lipid matrix or by degradation due to the enzymatic action, enhanced bioavailability, minimal drug absorption in blood vessels and high storage stability. The limitations can be listed as: the wastage of drugs while in fabrication, small loading capacity specifically for hydrophilic or polar molecules, the chance of drug expulsion from the lipid matrix as a result of polymorphic conversion while in storage, the inability to show robust release patterns and poor knowledge about clinical safety [85].

### 2.6. Nanostructured Lipid Carriers (NLCs)

NLCs emerged as the second generation nano lipid carriers to surmount the limitations of first generation nanocarriers. NLCs are nanocarrier systems derived from oil-in-water-type nanoemulsions. They are composed of lipids (physiological and biocompatible lipids), water and emulsifying agents/surfactants/co-surfactants. These are formulated by mixing solid lipids with small amounts of liquid lipids, which results in the rearrangement of the matrix structure. The components used in formulating the NLCs include oils (cetiol V, coconut oil), solid lipids (beeswax, palmitic acid, etc.), counter-ions (sodium hexadecyl phosphate, monodecyl phosphate) and emulsifying agents (egg lecithin, polyvinyl alcohol, etc.) [86]. NLCs and SLNs can be distinguished depending on this composition, i.e., if they have only solid lipids, they are called SLNs and when they have both solid and liquid lipids they are called as NLCs. Furthermore, due to the presence of a mixture of both lipids, a more amorphous structure is found which results in an improved loading capacity for lipophilic drugs as compared to SLNs. These can be easily prepared by three major techniques, which are the solvent-emulsification diffusion technique; double emulsion technique; and membrane contactor technique [86].

The advantages of NLCs can be listed as: a decreased polymorphic transition, little crystalline index, enhanced encapsulation efficiency, drug loading, physical stability, chemical stability and bioavailability, with the controlled release of the encapsulated components. Moreover, they can be altered with various targeting ligands such as peptides, antibodies or even small targeting moieties. Comparatively, the disadvantages include the irritation and sensitizing action of the surfactants; cytotoxic effects related to the nature of the lipid matrix and concentration; and the efficiency in the case of proteins, peptide drugs and gene delivery systems that need to be exploited [87]. Both SLNs and NLCs are considered suitable delivery systems for both skin and CNS distribution because of their aforementioned properties [88]. It is worth noting that NLCs have superior formulation properties (relatively more potent in controlling drug release and stability) over SLNs [85,89].

Both SLNs or NLCs having a particle size of less than 200 nm show good adhesiveness over the skin by forming a monolayer. This hydrophobic monolayer film exhibits occlusive action on the skin and hinders the loss of moisture owing to evaporation. This results in a reduction in the corneocyte packing and the opening of inter-corneocyte gaps, hence facilitating drug penetration into the deeper layers of the skin. The loss of water content from the SLN induces crystal modification of the SLN matrix and this can induce drug expulsion and penetration [90]. Moreover, the mechanism behind the enhanced drug bioavailability through the BBB could be explained by the surface modification of the SLN with Pluronic F-68. This results in a steric hindrance, which would further reduce the adsorption of opsonin onto SLN in the plasma therefore decreasing the RES uptake and delaying the plasma retention time. Further, a high load of SLN results in a higher concentration gradient at the brain capillary, resulting in enhanced transport across the brain endothelium following endocytosis and drug release [91].

### 2.7. Lipid Nanoemulsion (LNEs)

This has an oil-like lipid matrix and is usually prepared by incorporating drugs in the internal oil phase or at the interface of oil–water [92]. Thus, drugs with poor aqueous solubility can be loaded into the interior oil phase of a nanoemulsion, thereby reducing the hydrolytic reaction. These consist of submicron-sized lipid droplets, stabilized by surfactants that prevent accumulation and coalescence in an aqueous solution. LNEs can be prepared mainly by two broad techniques including high-energy methods (involving ultrasonication and high-pressure homogenization) and low-energy methods (involving phase inversion temperature and emulsion inversion point) [93]. LNEs were previously used for intravenously administered nutrients without having a drug carrier function, consisting mostly of plant-based lipid droplets with an average size of less than 500 nm [94]. These also serve as carriers for anaesthetic drugs, cancer therapeutics and vitamins and find applications in the pharma, cosmetics and food industries [95]. Homogenized milk and plant-based milk products can partly be considered as LNEs with additional protein ingredients.

These are also good vehicles for drug administration through the skin. LNEs are used to augment physicochemical properties that are desirable for transdermal drug delivery systems. They can enhance adhesiveness and hydration when they are applied over the skin. Nanosized fragments are able to form a thin film on the skin surface, thus enhancing the pore obstruction and hence reducing the water evaporation from the skin by maintaining skin hydration. LNEs are used for improving drug absorption through the skin, providing protection from degradation through the oral route, reducing irritation and offering a sustained release action [96,97]. These are also a promising system for delivering drugs directly into the brain (CNS targeting) through the intranasal route. Small (nano) droplets with a high surface area make them suitable for nose-to-brain delivery by avoiding the BBB. The possible mechanism for the transportation of drugs can be explained as transcytosis/endocytosis of the droplets by the brain endothelial cells through the nose. However, the presence of surfactant(s) in the formulation could have a fluidizing action on endothelial cell membranes, leading to improved drug permeability [98].

On the other hand, these have many factors that limit their uses, such as the use of ultrasounds or high-pressure homogenizers, the use of high concentrations of emulsifier, expensive production, the lack of knowledge about the mechanisms that affect and cause problems during their production (Ostwald ripening, i.e., the oil diffusion in the aqueous phase), etc. [99].

### 2.8. Microemulsion

Microemulsions are transparent, thermodynamically stable, colloidal drug carrier systems consisting of isotropic mixtures of oil, water and surfactant, often in combination with a cosurfactant. Usually, they have a droplet size of less than 100 nm and hence cannot be seen by the naked eye. Although microemulsions and nanoemulsions are completely different systems from the thermodynamic point of view, the size of the microemulsion may create confusion with a nanoemulsion. Microemulsions have a simple fabrication method, good stability, increased solubilization and bioavailability with a reduced cost which makes them suitable for practical applications [100]. They have the capability to incorporate both hydrophilic and lipophilic drugs which makes them advantageous over other delivery systems. They offer good solubilizing properties and improved drug permeation through biological membranes. Currently, these dispersions are used to deliver the drug through the skin and nasal membrane to reach blood and brains respectively [101,102,103]. Further, the smaller size of microemulsions can make their sterilization easy with a simple filtration method. Three distinct microemulsion systems that can be used in the pharmaceutical industry for drugs are oil in water, water in oil and bi-continuous microemulsions.

Microemulsions act as potential drug delivery systems through biological membranes. The efficacy of a microemulsion can be related to the improved solubilization as compared to conventional dosage forms. Further, the specific composition of microemulsions and the presence of surfactants and co-surfactants (may act as co-solvents for poor water-soluble drugs) can interact with the stratum corneum (as permeation enhancers) and enable the penetration of active ingredients through the skin [104,105,106]. Additionally, in the case of O/W microemulsions, oil droplets incorporating drugs can act as a reservoir, thus maintaining a high concentration gradient between the biological membrane and the applied formulation. The water causes increased hydration of the stratum corneum, thus improving the permeability [107].

## 3. Lipid-Based Nanocarrier System for Skin Delivery

The delivery of drugs across the skin can be used for achieving local (topical application) as well as systemic (transdermal application) actions. The local dermal delivery effect can be superficial, but for systemic delivery the release rate and penetration of the drug are significant factors for showing the therapeutic action. Transdermal drug delivery is an advanced route of drug conveyance for therapeutics activity, where the formulations containing the active ingredients are applied against the skin from where these molecules are absorbed slowly into deeper tissues. Subsequently, they either target receptors or enter into the systemic circulation and show the desired action. Transdermal delivery has several benefits, such as lower fluctuations of plasma drug levels, sustained or controlled drug release, circumventing first-pass metabolism, improving patient compliance compared to other delivery systems, etc. [108,109]. The skin acts as one of the essential routes for drug delivery by allowing topically administered drugs to travel through the stratum corneum [110]. Its wide surface area improves drug absorption and thus the higher quantity of drugs could be efficiently distributed through this. This route has become popular over the last 3 decades by providing several clinical advantages to patients [111]. Transdermal drug delivery systems (TDDS) can be classified into three generations: first generation TDDS includes drugs that are lipophilic, with a low molecular weight and an effectiveness at low concentrations; second generation TDDS yield additional advances for small-molecules delivery by increasing the skin permeability with the help of chemical enhancers, ultrasound or iontophoresis and act as driving forces for transdermal transport; and third generation TDDS facilitate the delivery of small-molecule drugs, macromolecules and virus-based and other vaccines through targeted permeabilization of the skin stratum corneum [112]. Traditionally, the transdermal route was only limited to lipophilic and non-ionic drugs, but the incorporation of nanotechnology into the transdermal route has opened the door for the delivery of hydrophilic ionic drugs as well. Further, the introduction of several lipid nanocarriers such as liposomes, niosomes, ethosomes, dendrimers and transferosomes for drug transportation not only boosts the degree of permeation but also minimizes the adverse effects with enhanced patient conformity [113,114].

Lipid-based nanocarrier systems have been developed to deliver the active moieties via several application routes, such as parenteral, skin, oral, ocular, nasal and pulmonary inhalation. Among them, skin delivery is one of the most investigated pathways for drug conveyance. It is well known that the human skin is a multifunctional organ. Besides other functions, it also acts as a delivery route for drugs by circumventing many of the limitations of oral and parenteral administration. The application of therapeutic products over the skin surface is one of the methods used for administering drugs. It is suggested that cutaneous administration of lipid-based nanocarrier systems is relatively safe by evading direct contact and also lessens the risk of acute and chronic toxicity. However, the barrier property of the skin restricts the effective permeation of the carriers which demands a safe and efficient system that provides an anticipated therapeutic action with minimal toxicity. Table 2 lists clinical trials relating to lipid carriers specifically crossing skin barriers. These indicate the clinical applications of the number of formulations for some diseases by using lipid carriers.

**Figure 2:**
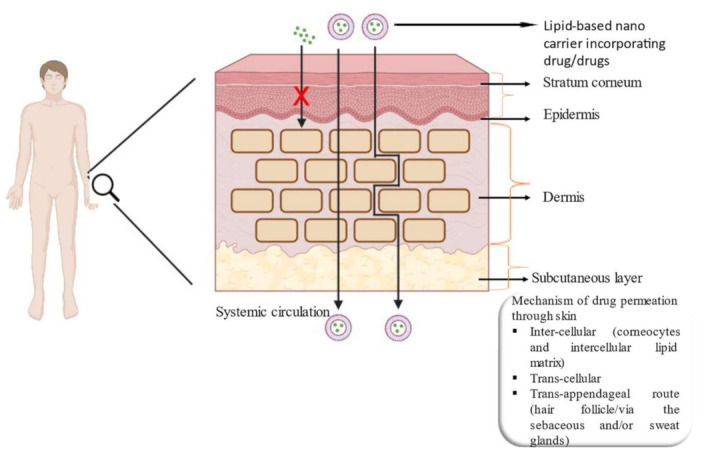
The structure of the skin depicting different mechanisms of drug permeation through it.

Drug delivery through the skin is a relatively safe method with reduced side effects as compared to other routes for treating diseases. The skin helps to mitigate the illness either by localized applications (dermal) or by systemic absorption (transdermal), depending upon the intended use. Skin administration avoids the hepatic first-pass effect and an enhanced localization of bioactive through skin organelles such as hair follicles. Lipid-based nanocarriers have been found to have the capability to treat diseases safely mainly due to the biocompatibility and the versatility of the lipids. Other advantages of this system may be listed as an improved drug stability, occlusion, skin hydration, penetration, retention and efficacy of the drug as compared to other drug nanocarriers. This type of delivery approach increases the concentration gradient in the upper skin layers and facilitates the gradual release from the system without causing cytotoxicity or morphological alterations in the skin layers. The various mechanisms by which a drug can pass through the skin can be listed as inter-cellular, trans-cellular and trans-appendages. The penetration mechanism through the skin depends on the physicochemical characteristics of free drugs (hydrophilicity/lipophilicity of the drug, the particle size and charge) and the drug delivery system [115] (its fluidity, stability and lipid content). The following Figure 2 depicts the structure of skin together with different mechanisms of drug permeation.

**Figure 3:**
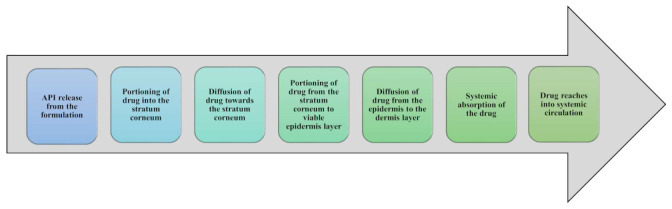
Representation of the different penetration pathways of the drug through the skin.

The proposed mechanism by which lipid carriers penetrate the skin involves making close contact with the superficial junctions of SC and diffusing between corneocyte islands, permitting the superficial spreading of the drug molecules. After application of the formulation, water evaporates [116], hence, the carriers form an adhesive occlusive layer over the skin. This occlusive layer increases the residence time and improves the skin penetration [117]. Additionally, hydration of the SC may reduce the packing of corneocytes, create gaps through corneocytes and affect the partitioning of the drug into SC. Lipid nanoparticles have the potential to deliver drugs via the epidermal surface (allow for lipid exchange between the SC and nanocarriers) as well as through follicles (vascular rich-reservoir) [118]. Furthermore, sebaceous glands present in the hair follicle secrete the triglycerides and waxes rich in sebum and squalene, making an environment enriched in lipids where carriers are entrapped. Furthermore, lipids present in nanocarriers may help the entry into the follicles. Additional evidence has been found that lipid-based nanocarriers are capable of interacting with lipid membranes causing lipid rearrangement, which can increase the penetration of encapsulated drug moieties [119]. Figure 3 gives a pictorial illustration of the penetration pathway of the drug into the systemic circulation through the skin.

The following paragraphs generate a proof of concept for depicting the usefulness of nano lipid carriers for delivering drugs across the skin both for local as well as systemic actions.

Some of the latest research elaborated upon the design and development of an ethosomal preparation incorporating Ranolazine (a newer novel anti-anginal drug) for a transdermal application. The objective of the study was to examine the potential of ethosomes as lipid nanocarrier vesicles for the transdermal enhancement of the model of a poor-soluble drug. The optimized ethosomal suspension showed a vesicular size of 165.71 ± 2.73 nm and zeta potential of −36.63 ± 2.78 mV. This release study demonstrated a superior sustained release over 24 h as compared to a plain gel incorporating Ranolazine. An MTT assay demonstrated increased cytotoxic potential of the drug on the H9C2-mouse cardiomyoblast cell line. In conclusion, the prospective ethosomal vesicles proved to have better safety, efficacy and patient compliance with minimum skin irritation through the transdermal route [120].

Another study demonstrated the formulation and evaluation of an NLC loaded with Olmesartan medoxomil an antihypertensive agent to overcome its poor bioavailability. The formulation was prepared by high-speed hot homogenization and the melt emulsification low-temperature solidification method and then was used as transdermal patches. The optimized NLC was found to possess a particle size, zeta potential and entrapment efficiency of 284 nm, −24.16 mV and 80.17%, respectively. The study concluded that the bioavailability of the drug was improved appreciably as compared to the pure drug and drug-loaded NLC for the oral route [121].

One recent study investigated the properties and function of a gel combining the *Centella asiatica* transfersomes and rosemary essential oil nanoemulsion. Here, this combination was used to synergistically prevent UVB radiation effects on the skin with the help of lipid-based nanocarriers for their anti-aging actions. The result showed that the size (less than 200 nm) and the physicochemical properties of lipid-based formulation increased the delivery of the active ingredients by overcoming the skin barrier. Further, the in vivo experiments revealed that the topical application of the gel could act synergistically and have the potential to prevent oxidative stress and collagen degradation in the skin from UVB-induced photoaging [122].

Another study developed and optimized potential NLCs embedded in transdermal patches for enhancing the transdermal delivery of capsaicin for skeleton–muscular and neuropathic pain. This research claimed that due to the exceptional properties and particle size (under 200 nm) of the lipid nanocarriers, capsaicin entrenched in the formulation penetrated faster in the epidermis and to a deeper extent than free capsaicin. These patches offered a sustained release and a superior accumulation in the deeper skin layers as compared to those containing free capsaicin in the form of conventional patches. Further, capsaicin NLCs-loaded patches exhibited comparatively lower skin side effects (in terms of skin irritation) than the conventional capsaicin patches, concluded from animal skin irritation experiments [123].

One of the investigations depicted the formulation and evaluation of naproxen transethosomal gel for sustained transdermal delivery for the management of musculoskeletal pain utilization. Transethosomes were prepared via the ethanol injection method and incorporated in carbopol 940 hydrogel. They were spherical in shape having a particle size in the range of 56.94 nm to 291.7 nm. The in vitro skin permeation study displayed an enhanced skin deposition and the in vivo studies showed the efficiency of naproxen transethosomal gel in lessening the oedema rate. The authors concluded that the developed formulation could be considered a perfect alternative to the conventional gel for managing musculoskeletal pain [124].

One more study was carried out on tocopherol succinate-loaded ethosomal gel for its delivery and evaluated its moisturizing, antioxidant and anti-aging effects. This formulation was prepared via the cold method and the particle size was found to be 179.1 nm with good entrapment. The in vitro release study showed an initial burst release pattern, the ex vivo permeation study showed increased drug accumulation into the layers of the skin and the in vivo study using a chronometer and cutometer demonstrated good moisturizing and mechanical properties, and good physical stability [125].

Another study formulated topical Apremilast-loaded NLCs gel for the management of psoriasis by using Compritol 888ATO, oleic acid, Tween and Span, and Transcutol P and finally dispersed in Carbopol. In vitro drug diffusion and ex vivo skin permeation results confirmed the potential of the NLC form of poorly water-soluble drugs for topical applications and improved drug deposition in the skin [126].

A different study investigated the simvastatin NLC-loaded transdermal patch prepared via the solvent evaporation method for its bioavailability enhancement, bypassing hepatic metabolism. Simvastatin is highly lipophilic but has been excreted very efficiently in an unchanged form after oral administration, making only 5% of drugs bioavailable for this therapeutic effect. This study proved that a transdermal patch showed an enhancement in the bioavailability of simvastatin as compared to the marketed oral dosage form, with an enhanced bioavailability and therapeutic effect [127].

In another investigation, a topical NLC formulation of the corticosteroid anti-inflammatory drug betamethasone dipropionate containing oleic acid was prepared to facilitate its penetration into the deeper layers of skin by overcoming systemic side effects. The NLC was prepared by high shear homogenization followed by the sonication method and was shown to penetrate more efficiently into the skin layers as compared to a traditional cream. Further, NLC formulations that comprised a higher percentage of oleic acid showed higher penetration through the skin [128].

One study described a novel topical SLN preparation of piroxicam for transdermal delivery with oleic acid as a penetration enhancer. The prepared SLNs have particle sizes is around 102 ± 5.2 nm with a zeta potential 30.21 ± 2.05 mV. It showed good compatibility between piroxicam and the vehicle components with a higher entrapment efficiency and sustained release pattern. The optimized SLN showed a quick anti-inflammatory action beginning in the 3rd hour after its application and proved to be a promising carrier for the encapsulation and sustained release of piroxicam, with great anti-inflammatory potential [129].

In one study, SLN, NLC and nanoemulsion of lornoxicam (LRX) were prepared by using Compritol^®^ 888 ATO, Lanette^®^ O and oleic acid for treating inflammatory conditions of the skin. All three formulations were compared in terms of their effectiveness against the said condition. They observed that all the formulations had a high drug payload, physical stability and drug-excipient compatibility. These formulations improved the skin penetration rate of the drug by three to four times as compared to a traditional gel incorporating LRX. Further, the nanoemulsion showed the highest rate of drug penetration through animal skin followed by NLC, SLN and a gel formulation. Thus, this study suggested all three formulations of LRX can be used topically for relieving painful and inflammatory conditions of the skin with decreased side effects compared to existing oral products [130].

Another study described the enhancement of the dermal retention and reduction in associated side effects of the poor water-soluble polyphenolic compound in azelaic acid for treating acne and rosacea by formulating NLCs. These were prepared via the solvent diffusion–solvent evaporation method for sustained release action. The mean particle size of azelaic acid-loaded NLCs was 81.57 ± 9.6 nm with a low PDI and high negative zeta potential. The authors concluded that the developed NLCs have enormous potential to improve the penetration of azelaic acid through the skin, which is the requirement for the topically applied formulations for the management of skin diseases by avoiding systemic adverse effects associated with its usage [131].

From the above-cited research, we can conclude that lipid-based nanocarriers are widely used for both the local and systemic delivery of drugs across the skin. They display relatively greater effectiveness as compared to free drugs with sustained action, good stability, compatibility and fewer skin side effects. They also avoid systemic adverse effects associated with oral dosage forms with improved patient compliance. Additionally, these lipid-based systems also show good effectiveness for poorly water-soluble drugs as evident through various in vitro, ex vivo and in vivo studies.

## 4. Lipid-Based Nanocarrier Systems for CNS Delivery

The BBB is the primary obstruction for CNS delivery by controlling the influx and efflux of biological substances. This is a highly specialized structure composed of microvascular endothelial cells covering the cerebral capillaries penetrating the brain and spinal cord of most organisms. Brain microvascular endothelial cells control exchanges between the blood and surrounding cells but provide limited diffusion. This is because these cells assembling the microvascular lining of the BBB have no or less gap in the outer membrane. Along with these cells, the BBB also consists of two other cellular components including pericytes and astrocyte end-feet that combine with neuronal terminations to form the BBB [132,133]. The cerebral endothelial cells combine with astrocytes and mediate signals that help in the formation and maintenance of the tight junction, which specifically excludes most of the blood-borne materials into the brain. In the mammalian brain, astrocytes are not believed to have a barrier role, but the astrocytic end-feet tightly confine the vessel wall and play a critical role in the induction and maintenance of the tight junction [18]. Pericytes occupy the perivascular space, between the capillary wall and astrocytes’ end-feet, except in the large vessels and take part in controlling the vasculature tone, repair and stability [134,135]. Further, pericytes have limited angiogenesis and provide microvascular stability by reducing the growth of capillaries. They regulate capillary diameter by their contractile function and therefore affect the oxygen and nutrient diffusion. Lastly, neuronal terminations arrive at all cells forming the BBB [136]. Generally, neurons are closely connected with capillaries, maintain microvascular permeability and control blood flow. It also regulates angiogenesis by different releasing factors and helps with the synthesis and localization of tight junction molecules in the brain. Further, microglia and leukocytes are other cells that aid in maintaining the integrity of the BBB. They produce an inflammatory response against infection, stress and other alterations in the brain.

Despite advanced CNS drug discovery, the majority of the CNS disorders remain undertreated because of the lack of an effective drug delivery system. It is well accepted that the failure of therapy is attributed not only to the potency of the drugs, but also to the presence of barriers that hinder the efficient delivery of the molecule (such as BBB in the case of the brain). Additionally, large molecular mass hydrophilic molecules carrying charge further exaggerate the problems because of the very poor diffusional properties in the biological milieu. However, the poor aqueous solubility and low potency of therapeutic agents in the CNS lead to higher toxicity and major side effects, demanding potential delivery models for brain treatment. Table 3 lists clinical trials involving pharmaceutical formulations administered through transdermal and intranasal routes, specifically for brain disorders.

Generally, to enter the brain drugs should pass through the endothelial cells. Primarily there are two passages for this purpose that can be described as: (1) the paracellular pathway (between endothelial cells) which could be used for the transportation of ions and solutes crossing the BBB, and (2) the transcellular pathway/transcytosis (on the endothelial cells) which could be used for the transportation of many substances across the BBB. Normally, the transcellular pathway allows for the passive diffusion of lipophilic molecules with a size of less than 500 Da. It further allows for the transportation of gas molecules depending on specific receptors, the transportation of hydrophilic polar molecules such as glucose and proteins depending on specific transporters (e.g., GLUT-1, choline transporters and large-amino acid transporter 1 or specific receptors for transferrin, insulin, lipoprotein and interleukin-13). These mechanisms could be employed to design nanomaterials for crossing the BBB. Caveolae is another transportation pathway in the brain which depends on a formed vesicle around the molecules through cellular invagination. Currently, the transcellular pathway has been widely investigated and several approaches have been devised for the transportation of drugs into the brain. Nonetheless, the active efflux pumps lessen the BBB-crossing efficiency of most of the drugs [137]. The system plays very crucial functions in maintaining the normal physiological environment of the brain by eliminating heterologous materials and toxic metabolites, so this could be downregulated by enzymes as per the disease’s demand [138].

**Figure 4:**
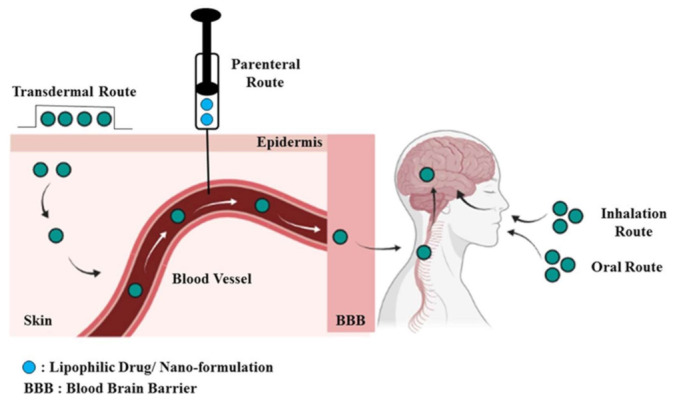
Different routes of administration through which drug reaches to brain by crossing BBB.

**Figure 5:**
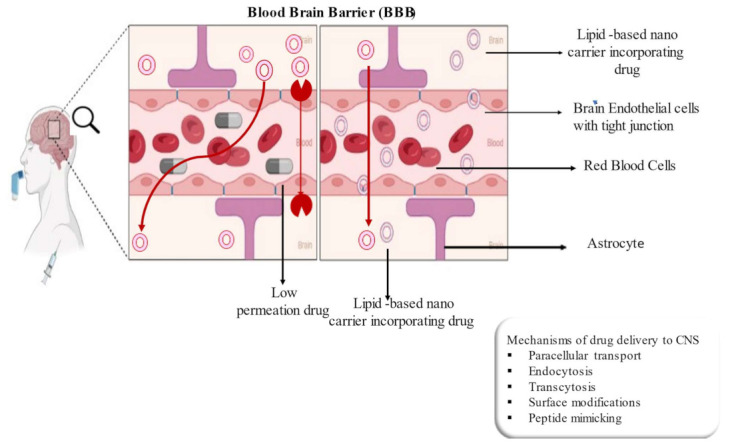
Structure of BBB showing the different mechanisms of drug delivery across CNS.

Nanocarriers deliver the drugs into the CNS either directly after peripheral administration or alternatively through the naturally occurring trans-synaptic retrograde passage to enter the CNS from the peripheral nerves as described above. The intranasal route is another route by which a drug has directly entered the brain after intravenous administration by bypassing the BBB [139,140,141]. Figure 4 shows the different routes of administration through which the drug reaches the brain by crossing the BBB. It has been proved that the identification and interaction with the receptors present on the endothelial cells of the brain capillary are primarily responsible for the brain uptake of the drug. The suitable modification of the surface of the nanocarriers improves their circulation time in the blood, thus decreasing their uptake into the reticuloendothelial system (RES) organ [142]. Endocytosis through the low-density lipoprotein (LDL) receptor of the endothelial cells after the adsorption of lipoproteins to the nanoparticle is the mechanism by which these carriers move across the BBB. Further, the prevention of binding to cellular efflux systems such as p-glycoprotein may increase the brain uptake of drugs by preventing their removal [143]. Receptor-mediated transcytosis through the BBB provides the potential for high selectivity in brain targeting [144]. Monoclonal antibodies are suitable carriers for this purpose as they mimic peptide structures thus deemed useful for targeting [145]. Alternatively, the osmotic opening of the cerebral capillary by using the pump is another advanced technology which is used to open the BBB and target the drug to the CNS. The following Figure 5 shows the structure of the BBB and illustrates different mechanisms by which the drug delivers across the CNS.

Lipid-based nanocarrier systems that are primarily comprised of natural, biocompatible materials and/or a combination of natural lipids may provide a solution for this problem. The size of these systems lies in the nano range which makes them flow in the systemic circulation, preventing macrophage uptake. The composition of these systems is generally similar to that of cell membranes hence, they have a minimum impact on both the intracellular and extracellular environments [22,88]. Further, they increase the drug retention time in brain capillaries by maintaining a concentration gradient of drugs between the blood and brain tissues, opening tight junctions to facilitate movement through the BBB and transcytosis of drug-loaded lipid carriers through the endothelium layer [85,146]. Owing to the above-mentioned advantages, lipid-based nanocarriers act as a promising approach for managing various brain disorders as compared to inorganic and polymer-based systems [147,148]. In this section, current research related to lipid nanocarriers that generally target the brain and related disorders through different routes of administration is discussed, which generates proof of research and development in these areas with prospective directions for novel interventions.

Recent research describes the preparation and characterization of valproic acid NLC via the high-speed homogenization method through the nasal route of drug administration bypassing the BBB. The particle size of the NLC was found to be 142.5 nm which is in the optimal range for BBB permeation. The rate of release of Valproic acid from the optimized NLC was found to be 66.12% in 3 days and permeations of 26.55% in 6 h. The study results indicate that this NLC is efficient in being absorbed through the nasal route and delivering the drugs at a therapeutically sufficient concentration into the brain for treating epilepsy without causing damage to the BBB [149].

Recent experiments designed and developed a transferrin-decorated NLC containing rapamycin. This drug has low water solubility, rapid in vivo degradation and an immunosuppressive effect. Further, the BBB-related challenges limit its clinical use for brain diseases. Here, NLCs were prepared to have particle sizes ranging from 120 to 150 nm and a good entrapment efficiency and showed ≥80% cell viability in the MTT assay. They displayed a significantly increased cellular uptake (97% vs. 60%) after 2 h of incubation and an appropriate brain accumulation with a lower uptake on U-87 MG glioblastoma cells. Moreover, rapamycin-loaded NLCs did not exhibit an immunosuppressive effect. In conclusion, Tf-decorated NLCs can be considered a safe and efficient carrier for the brain-targeting of rapamycin for treating neurological disorders [150].

One latest study developed a targeted delivery system based on an NLC modified with two proteolytically stable D-peptides, D8 and RI-VAP called Dual-NLCs. D8 holds a high affinity towards nicotine acetylcholine receptors and can penetrate through the BBB efficiently. RI-VAP is a specific ligand of the cell surface GRP78 and a specific angiogenesis and cancer cell-surface marker, capable of circumventing the blood-brain tumor barrier (BBTB). The study showed that this formulation could penetrate through in vitro BBB and BBTB models with excellent efficiency and could internalize into brain capillary endothelial cells, tumor neovascular endothelial cells and glioma cells with high specificity as compared to non-targeted/mono-targeted NLCs. Maximum therapeutic efficiency was established by an in vitro cytotoxicity study. Further, the superior targeting capability and the efficient anti-glioma behavior were confirmed through an animal study. Conclusively, this formulation has the potential for advanced brain cancer treatment with promising results [150].

Another current study explains the berberine-loaded NLCs coated with chitosan (BER-CTS-NLCs) via the intranasal route for treating Alzheimer’s disease. The size of the formulation was found to be 180.9 ± 4.3 nm having sustained-release properties, a surface charge of 36.8 mV and improved ex vivo permeation through nasal mucosa It has been found that the intranasal formulation of BER-CTS-NLCs is a more efficient system for maintaining greater drug levels in the brain with increased drug targeting efficiency as compared to a BER solution. Authors concluded that the optimized NLCs are speculated to be a successful approach for improving the action of BER in treating Alzheimer’s disease through the proposed route [151].

In one more study, Riluzole-loaded NLCs were formulated and functionalized with lactoferrin (Lf), which improved conveyance within the BBB by interacting with Lf receptors in the endothelium of the brain to treat the neurodegenerative disease Amyotrophic lateral sclerosis. Nanoparticles exhibited a particle size in the range of 180 and 220 nm, a narrow size distribution and at least 3 months of stability. The encapsulation efficiency was found to be 94–98%. By formulating riluzole-loaded NLC, the brain targeting increased by overcoming the BBB. Further, the authors claimed the biocompatibility of the formulation through an MTT assay and the stability of the system for 3 months with the suitability of the delivery system for the brain [152].

In a current investigation, omega-3 (ω-3) fatty acid nanoemulsions (NEs) loaded with curcumin (CUR) and quercetin (QU) were prepared and claimed to have improved treatments efficiency, as they had the potential to increase the nose-to-brain permeation of CUR and QU. These compounds possess good anti-inflammatory and antioxidant properties but have low water solubility and poor bioavailability, hence the nanoemulsion was designed to increase these limitations. The study claimed the stability and sustained release behavior of the system as described above for the efficient treatment of neurodegenerative brain diseases. Furthermore, the toxicity of the formulations was tested in an animal model, *C. elegans*, and the results demonstrated no toxicity for all tested formulations in varieties of concentrations [153].

Another study described the thymoquinone-enriched naringenin (NGN)-loaded NLC through the nasal route for mitigating depression. Here, Thymoquinone (TQ) oil was used as the liquid lipid and showed synergistic actions. The droplet size was found in the range of 84.17 to 86.71 nm with narrow PDI and good entrapment efficiency. The study proved that the release profile, permeation, antioxidant and pharmacodynamic behavior of the TQ-loaded NGN-NLC was better as compared to the NGN suspension through the nasal mucosa. In conclusion, the TQ-enriched NGN-NLC was proved to be an efficient system for the brain delivery to abate depression [154].

One of the studies claimed a delivery system with macrophage (MA) membrane-coated SLNs on the surface of the MA membrane, created by binding the rabies virus glycoprotein (RVG29) and triphenylphosphine cation molecules for efficient antioxidant delivery to neuronal mitochondria. According to the investigation, the MA membranes masked the SLNs and prevented elimination via RES-rich organs by inheriting the macrophage’s immunological characteristics and helping to cross the BBB and selectively targeting neurons. This was possible owing to the presence of RVG29 on the surface. This Genistein-encapsulated DDS demonstrated the most favorable outcomes in relieving Alzheimer’s disease symptoms in vitro and in vivo via the synergistic action of the combination of MA membranes RVG29 and TPP [155].

Another finding describes the treatment of Alzheimer’s disease by repurposing imatinib mesylate-loaded liposomes which were originally used as an anti-cancer agents. Liposome was fabricated to overcome the side effects and poor brain bioavailability of the drug. The particle size was optimized up to 150 nm with a sustained-release action up to 96 h. The authors claimed that there was improved brain deposition and enhanced residence time via the nose-to-brain administration for the liposomal formulation in comparison with simple drug solutions through pharmacokinetics study [156].

Current research compared the poly (lactic-co-glycolic acid) nanoparticles (PLGA NPs) and SLNs for the intranasal delivery of meloxicam. The authors observed that SLNs displayed an increased encapsulation efficacy (EE) and drug loading (DL), mucoadhesion, permeation and in vitro release than particulate NPs. They claimed that there was improved brain deposition of the SLNs through animal experiments. In conclusion, the MEL-encapsulated C-SLNs are a promising carrier in enhancing the brain bioavailability of meloxicam through the intranasal route as compared to PLGA NPs [157].

Another investigation explained the SLN of Rose damascene prepared via the micro-emulsion method with covalently attached lactoferrin onto the surface of the nanoparticles for targeted delivery. Rose damascene may increase memory and acetylcholine esterase inhibition but simultaneously has a hydrophilic content, and hence cannot pass the BBB. For this reason, SLNs were fabricated with a lactoferrin coating. The results revealed that the size, entrapment efficiency and drug loading were found to be 52 nm, 98, 93.6 before conjugation and 161 nm, 11.2, 15.9 after conjugation, respectively The pharmacodynamics study result showed that the formulation can improve short-term memory and may also decrease anxiety and depression in scopolamine-induced animals [158]

In a new study, Dopamine-SLN adsorbing proanthocyanidins (GSE/DA-SLN) were formulated by combining Dopamine (DA) and proanthocyanidins (antioxidant derived from grape seeds) for treating the oxidative stress related to Parkinson’s disease. The objective of the study was to control the missing DA and to increase the levels of the neurotransmitter that is needed for a patient suffering from Parkinson’s. However, these were unable to cross the BBB, hence an SLN was fabricated. Proanthocyanidins were found on the SLN surface and had efficacy in preventing DA autoxidation. They showed high mucoadhesive strength, no cytotoxicity through the MTT test and were claimed as an effective nose-to-brain delivery system for Parkinson’s disease [159].

Another experiment explained the formulation of SLNs encapsulating Rutin for the effective brain delivery to target tumors through the BBB. Rutin-loaded SLNs were fabricated via the oil-in-water microemulsion technique, producing spherical-shaped particles with an average particle diameter of 100 nm and having stability for 5 days in circulation. In vitro studies proved the biocompatibility and stability of the formulation. The study concluded that the Rutin-encapsulated SLN formulations can have the capacity to target tumors across the BBB [160].

One of the researchers described the development of a stable NLC system for carrying curcumin for brain cancer therapy. The formulation had a particle size of 146.8 nm, zeta potential of −21.4 mV and entrapment efficiency of 90.86%. This curcumin-loaded NLC showed increased cytotoxicity to the astrocytoma–glioblastoma cell line than simple curcumin. Further, the biodistribution studies showed higher drug concentrations in the brain after the intranasal administration of NLCs than that of the plain drug and proved to be a promising drug delivery system for brain cancer therapy [161].

This current study describes the design, development and performance of the ligustrazine phosphate transdermal ethosomal system to treat Alzheimer’s disease for its antioxidant effect. The study result showed that the penetration ability, as well as the drug deposition in the skin of the ethosomal formulation, were significantly higher than those for the aqueous one. Further, this transdermal ethosomal system improved the behavioral performance in the animal models and offers a potential alternative therapeutic drug in the fight against Alzheimer’s [162].

The present research described the formulation of nanosized drug delivery systems using liposomes and niosomes incorporating pramipexole for Parkinson’s disease. Furthermore, it evaluated the penetration and antiparkinsonian action of the nanosized, PEGylated pramipexole-encapsulated liposomes and niosomes. The in vitro evaluation for penetration using the BBB cell co-culture model for all formulations was also done. The in vivo effectiveness was also tested. The results showed that all formulations were BBB-permeable and displayed better effects at a lesser concentration than that of conventional pramipexole tablets used for routine treatments. The study concluded that the nanosized pramipexole-encapsulated neutral niosomes showed good therapeutic effects in animal models [163].

This study investigated the niosomes incorporating the oral alkylating agent temozolomide with chlorotoxin surface modification for targeting glioblastomas. Surface modification with the peptide made it a highly potent system to target the glioblastoma cells of brain tumors. Temozolomide-loaded niosomes were formulated using the conventional thin-film hydration method, characterized and evaluated. The particle size of the chlorotoxin-loaded formulation was found to be 220 ± 1.45 nm with an entrapment efficiency of 79.09 ± 1.56%. Quantitative tissue distribution studies revealed that improved penetration of the drug into the brain may have been owed to the surface modification, with fewer depositions in the highly perfused organs [164].

The current research was an attempt to systemically deliver a schizophrenia drug, Quetiapine Fumarate (QF), through a non-invasive intranasal route using NLC to overcome the challenges of the BBB. The desired QF-loaded NLCs were developed and monitored to improve the QF bioavailability and brain targeting abilities. The result showed that there was no morphological damage after administration. Further, the ex vivo study displayed a two-fold increase in the ex vivo nasal diffusion as compared to QF. The QF blood–brain ratio showed a 10-fold increase for NLCs administered through the nasal route (in comparison to the intravenous route), supporting retention of the drug at the desired site. The study supports a potential nose-to-brain transport of QF-NLC for the effective management of schizophrenia by circumventing the BBB [165].

The above-mentioned studies described the capabilities of lipid-based nanocarriers for ameliorating brain disorders through different routes either by crossing or by circumventing the BBB. They support the retention of the drug at the desired site with enhanced residence time and no morphological damage. Further, they proved to be better than their conventional counterpart in relation to their bioavailability and therapeutic efficacy. These investigations might encourage researchers to investigate their future applications in this arena.

## 5. Conclusions and Future Prospective

Currently, lipid-based nanocarriers provide an outstanding potential and use as highly competent and safe treatment options by conquering the barriers of skin and BBB. The application of lipid nanoparticulates in skin drug delivery systems is increasing recently as they are safe, biocompatible, biodegradable, and have satisfactory pharmacokinetics and pharmacodynamics properties with enhanced benefit–risk ratios. They are efficient at providing protection against the degradation on the skin’s surface, creating a concentration gradient in the upper layer of skin and facilitating steady release. Further, they do not cause cytotoxicity or morphological alteration in the skin layers. The same effect can also be achieved for brain disorders by utilizing lipid-based nanocarriers, as they hold a promising natural tendency to distribute the drugs directly to the brain on account of their lipidic nature. Although they have potent applications to target the brain by crossing the BBB, the optimization of the dose should be accomplished to achieve an optimal therapeutic index. Along with the screening and stability of the lipid in the bloodstream, surface functionalization should also be investigated to limit the accumulation in normal tissues and/or the BBB. Further, limited evidence discloses the ability of lipids for brain delivery after oral administration by achieving sufficient therapeutic windows to enhance the permeability of the BBB. However, there are gaps in the literature regarding the form and concentrations of lipids required to produce the anticipated outcome. The use of lipid carriers for brain targeting through the oral route has not been investigated fully to determine with certainty if similar permeability-enhancing effects would be observed for parenteral administration, hence demanding a proper investigation. The physiological lipids and excipients that are used for fabricating these types of formulations are generally recognized as safe [166] and the majority of the components are also approved for use in pharmaceutical formulations. However, targeting ligands linked with main molecules may sometimes be responsible for producing toxicity, hence, demanding evaluation before marketing these products [167]. Further, genotoxicity and hemocompatibility studies may provide positive results for the successful market launch of these carriers. At present, most investigations are still only in the preclinical or early clinical stages, so due to the limited results, they cannot yet reach a definitive conclusion, suggesting further investigation using the lipid nanocarriers in suitable preclinical and clinical models. This will definitely help to extend their applications futuristically for several other purposes.

## Figures and Tables

**Table 1:** Different marketed formulations incorporate lipid carriers for various disorders.

S. No.	Type of Lipid Carriers (Delivery System)	Name of the Marketed Formulation	Active Ingredients	Advantages of Used Delivery System	Advantage/ Uniqueness	Route of Administration	Reference
1.	Liposome	Liposomal Glutathione: Livon Labs	d-Lenolate Olive Leaf Extract, d-Lenolate Pain Serum, d-Lenolate Probiotic 10 Billion	Increases absorption of glutathione by decreasing oxidative stress biomarkers. Increases immune function markers with the liposomal delivery system	Powerful antioxidant, anti-aging property, protect liver and remove the excretory waste from the human body	Oral	[42]
		California Gold Nutrition Liposomal Vitamin D3	Liposomal Vitamin D3	Increases absorption and hence increases bioavailability	Increases bone health and immunity	Oral	[43]
		Aurora Nutrascience	Mega-Liposomal NAD+/Resveratrol, Organic Fruit	Increases bioavailability	Increases longevity, increases cognitive and neurological function, increases energy and healthy muscle function	Oral	[44]
		MPC-Liposomes	Milk peptide complex	Liposomes are used to entrap MPC (Milk Peptide Complex) for increasing permeation	Regenerating and anti-aging agent, advised for skin regeneration and for prevention of skin damage due to the aging process	Topical	[45]
		Sesderma C-VIT Facial Liposomal Serum 30 ml	Ascorbyl glucoside encapsulated in liposomes, mulberry extract, liposomed hyaluronic acid, Syncoll and Panthenol	Encapsulated vit-C in liposomes and hence increases stability. Increases penetration due to structure analogous with the biological membranes.	Offering facial luminosity and vitality	Topical	[46]
		BodyBio PC liposomal phospholipid complex	Phosphatidylcholine	Liposomal system contains pure phospholipids which are not broken apart and are instantly utilized, re-building every cell in your body as compared to non-liposomal PCs, which are broken down during digestion	Increased memory, clarity, focus and cell repair	Oral	[43]
		AmBisome	Amphotericin	Liposomal encapsulation can substantially affect drug’s functional properties relative to those of the unencapsulated drug, decreasese the size & increases the bioavailability	Fungal infection in febrile, neutropenic patients	Parenteral	[47]
		DEPOCYT- Enzon Pharmaceuticals Inc., North Bridgewater, NJ, USA	Antimetabolite cytarabine	Sustained-release formulation	Symptoms of Acute Nonlymphocytic Leukemia, Meningeal Leukemia, Refractory Leukemia, and Lymphomatous Meningitis	Parenteral	[47]
		Estrasorb- Novavax, Rockville, MD, USA	Estradiol	Increased efficacy with a lesser dose	Hot flashes and symptoms of Vulvar and Vaginal Atrophy related to menopause	Topical	[48]
2.	Niosome	LEP-ETU: Neopharma	Paclitaxel	Decreases toxicity, while increasing the efficacy	Anti-cancerous, used in advanced cancer treatment	Parenteral	[49]
		Lancome^®^—Loreal, Paris, France	10% Bifidus Prebiotic Grand Rose Extracts, Vitamin E, Pro-Xylane, and a gentle Exfoliating Acid Complex	Increases stability and increases penetrating power through the skin	Anti-aging agent; increases epidermal cell renewal to obtain younger-looking skin	Topical	[50]
3.	Nano emulsion	Restasis^®^: Allergan	Cyclosporine A	Decreases size, increases ocular penetration, provide sustain release action and decreases side effects	Lubricate eyes	Ophthalmic	[49]
		Hanacure: Nano Emulsion Multi-Peptide Moisturizer	Peptides, squalane, sodium hyaluronate and mushroom extract	Decreases size, increases penetration	The moisturizer intensifies the anti-aging effects and increases the appearance of the skin’s tone and texture	Topical	[43]
		Coolnac Gel Emulgel 1%—Chumchon	Diclofenac diethylammonium	Decreases size, increases penetration	Anti-inflammatory gel	Topical	[51,52]
		Ketopatch 30mg transdermal patch	Ketoprofen [30mg]	Decreases size, increases penetration	Anti-inflammatory action	Topical	[47]
4.	Solid lipid nanoparticles	Under clinical trial	Rifampicin + isoniazide	Decreases dosing frequency and increases patient compliance for better management	Anti-TB agent	Inhalation	[49]
		Cutanova Cream Nano Repair Q10—Dr. Rimpler	Q10, polypeptide, hibiscus extract, ginger extract, keto sugar	Increases moisturizing properties, increases consistency and spreadability	Anti-aging agent	Topical	[53,54]
5.	Nanostructured lipid carriers	NLC Deep Effect Eye Serum: Beate Johnen	Coenzyme Q10, a highly active oligosaccharide	Deeper penetration	Remove dark circles, anti-aging, rejuvenate skin	Topical	[55]
		Dr. Rimpler Cutanova Cleanser	Soluble Collagen, Tocopherol, Helianthus Annuus Seed Oil	Prevent oxidation and permit controlled release	Skin cleanser without removing valuable skin lipids and maintains the moisture balance of the skin	Topical	[43]
		NanoLipid Restore CLR™ by CLR Berlin	The liquid suspension containing 27% black current seed oil	Prevent oxidation and permit controlled release	Forms a film on the skin due to its physical properties, thus providing an excellent skin feeling. It is designed for regenerative care of dry, rough, scaly and aged skin	Topical	[47]
6.	Ethosome	Nanominox—Sinere, Germany	4% minoxidil, adenosine, sophora flavescens extract, creatine ethyl ester, cepharanthine, B12, ethanol	Increases skin delivery	Baldness	Topical	[56]
	Transethosomes	Ketoprofen transdermal—IDEA AG	Ketoprofen	Decreases size and increases skin permeation	Musculoskeletal pain	Topical	[43]

**Table 2:** Clinical trials relating to lipid carriers specifically crossing skin barriers.

S. No.	Study Title	NCT No.	Volunteer/Actual Enrolment	Recruitment Status	The Phase of the Clinal Trial	Treatment Intervention	Clinical Trial Sponsor	Country
1.	Formulation and Clinical Evaluation of Ethosomal and Liposomal Preparations of Anthralin in Psoriasis	NCT03348462	20	Completed	4	Psoriasis Vulgaris	Assiut University	Egypt
2.	Proof-of-Concept Study of Topical 3%-Diclofenac-Nano-Emulsion Cream for Knee OA Pain	NCT00484120	123	Completed	2	Osteoarthritis of the Knee	Pharmos	Israel
3.	Clinical Assessment of Oxiconazole Nitrate Solid Lipid Nanoparticles Loaded Gel	NCT03823040	28	Completed	1	Tinea	Minia University	Egypt
4.	The Study of Efficacy and Safety of a 60-day Use of the Magnox Comfort Compared to the Placebo in Subjects With NLC	NCT03807219	216	Completed	NA	Nocturnal Leg Cramps	Naveh Pharma LTD	Ukraine
5.	Liposomal Lidocaine Gel for Oral Topical Anesthesia	NCT01425840	40	Completed	1	Anesthesia	University of Campinas, Brazil	Brazil
6.	Transdermal Testosterone Nanoemulsion in Women Libido (Biolipid/B2)	NCT02445716	70	Unknown	2	Menopause	University Potiguar	Brazil
7.	NB-001 Treatment of Recurrent Herpes LabialisNanoemulsion	NCT01695187	362	Unknown	3	Herpes Labialis	NanoBio Corporation	USA
8	Efficacy of Topical Liposomal Form of Drugs in Cutaneous Leishmaniasis	NCT01050777	30	Completed	Early Phase 1	Cutaneous Leishmaniasis	Tehran University of Medical Sciences	Iran, Islamic Republic of

**Table 3:** Clinical trials involving pharmaceutical formulations administered through transdermal and intranasal routes, specifically for brain disorders.

S. No.	Study Title	NCT No	Volunteer/Actual Enrolment	Recruitment Status	Phases of Clinical Trial	Treatment Intervention	Clinical Trial Sponsor	Country
1.	Efficacy of Transdermal Nicotine, on Motor Symptoms in Advanced Parkinson’s Disease (NICOPARK2)	NCT00873392	40	Completed	2	Idiopathic Parkinson’s Disease	Assistance Publique—Hôpitaux de Paris	France
2.	Efficacy and Safety of Rivastigmine Transdermal Patch in Patients with Mild to Moderate Alzheimer’s Disease	NCT00423085	859	Completed	3	Alzheimer’s Disease	Novartis Pharmaceuticals Ono Pharmaceutical Co. Ltd.	Japan
3.	The Efficacy and Tolerability of NP101 Patch in the Treatment of Acute Migraine (NP101-007)	NCT00724815	530	Completed	3	Migraine Disorders	NuPathe Inc.	USA
4.	Study to Evaluate Safety & Efficacy of d-Amphetamine transdermal System Compared to Placebo in Children & Adolescents With ADHD	NCT01711021	106	Completed	2	ADHD	Noven Pharmaceuticals, Inc.	USA
5.	Phase I Open Label Single-Dose Study to Compare the Pharmacokinetics of NP101 Healthy Volunteers (NP101-006)	NCT00720018	4	Completed	1	Migraine Disorders	NuPathe Inc.	USA
6.	Treatment of Parkinson’s Disease with a Transdermal Skin Patch	NCT00001931	20	Completed	2	Parkinson Disease	National Institute of Neurological Disorders and Stroke (NINDS)	USA
7.	Does Topical Steroid Treatment Impair the Adrenal Function?	NCT00476489	50	Unknown	NA	Hypothalamus–Pituitary–Adrenal Axis Assessement	HaEmek Medical Center, Israel	Israel
8.	Efficacy of Euminz^®^ for Tension-Type Headache (CAS/B/016611)	NCT01770080	211	Completed	4	Episodic Tension-Type Headache	Charite University, Berlin, Germany Cassella-med GmbH & Co. KG	Germany
9.	Study of Intranasal Clonazepam in Adult Subjects with Epileptic Seizures	NCT00594945	45	Completed	2	Epilepsy	Jazz Pharmaceuticals	USA
10.	Effect of Intranasal Oxytocin on Headache in Chronic Daily Headache	NCT00963040	40	Completed	NA	Chronic Daily Headache	MedVadis Research Corporation	USA
11.	TI-001 (Intranasal Oxytocin) for Treatment of High-Frequency Episodic Migraine and Chronic Migraine	NCT01839149	240 participants	Completed	2	High-Frequency Episodic Migraine and Chronic Migraine	Trigemina, Inca	Australia
12.	Intranasal Civamide for Episodic Cluster Headache	NCT00069082	2	Completed	3	Episodic Cluster Headache	Winston Laboratories	USA
13.	Study of Nasal Insulin to Fight Forgetfulness—Long-acting Insulin Detemir—21 Days (SNIFF-LONG 21)	NCT01547169	60	Completed	2	Alzheimer’s Disease and mild Cognitive Impairment	University of Washington National Institute on Aging (NIA)	USA

## Data Availability

Not applicable.

## Supplementary Materials

The following supporting information can be downloaded at: https://www.mdpi.com/article/10.3390/membranes13030343/s1, Table S1. List of patents showing specific lipid-based carriers that are enabled to cross the skin and brain barriers.
