# Supplementary material for: Potential of Lipid-Based Nanocarriers against Two Major Barriers to Drug Delivery—Skin and Blood–Brain Barrier
Source: Membranes (Basel). 2023 Mar 16;13(3):343. doi: 10.3390/membranes13030343 (PMC10058721; doi:10.3390/membranes13030343)
Supplement: Supplementary file 1 [file membranes-13-00343-s001.zip › membranes-2188925-supplementary.pdf]

Table S1. List of patents showing specific lipid-based carriers that are enable crossing of the skin and brain barriers.

S. No.	Patent No.	Title	Applications	Description	Targeted Biological Barrier
1	CN102579323A	Paclitaxel ethosome gel and preparation method thereof	Reduces the skin stimulation and improves transdermal penetration	Contains paclitaxel, phospholipid, cholesterol, low-molecular-weight alcohol, stabilizing agent and antioxidant in a gel matrix, with penetration accelerator, humectant, preservative, pH conditioning agent and water	Skin
2	CN106955277B	Transdermal drug delivery system of alcohol liposome containing hyaluronic acid and preparation method and application thereof	TDDS of a liposome having hyaluronic acid, its preparation method and application	Liposomal formulation in the fields of external preparations, cosmetics and the like, especially as a drug delivery vehicle for topical skin external administration	Skin
3	CA2656438A1	Nanostructured lipid carriers containing riluzole and pharmaceutical formulations containing said particles	Treatment of amyotrophic lateral sclerosis and multiple sclerosis	Nanoparticles comprising drugs entrapped in lipids	Brain
4	CN102614091B	Resveratrol nanostructured lipid carrier and preparation method thereof	Preparation method for resveratrol-containing cosmetics	The resveratrol nanostructured lipid carrier has good stability and water-solubility with an easy and reproducible preparation method	Skin
5	WO2014144365A1	Nano-liposomal formulations and methods of use	Method for transdermal drug delivery for the topical administration of liposome encapsulated nanoparticles	Incorporating an agent in liposomal vesicles to form number of encapsulated nanoparticles having required size and topically administering these encapsulated nanoparticles for passive transdermal delivery of the agent to a patient	Skin
6	KR20080038812A	A cosmetic of sheet-type containing insoluble material	Cosmetics that increase the effect of insoluble material to the skin	A cosmetic composition that includes a sheet where a low-viscous nano-emulsion containing a liposome	Skin

		stably in a low viscosity level state	by increasing dispersion property	insoluble material is immersed, and the insoluble material is at least one selected from the following group: retinol, a retinol derivative, tocopherol, tocopheryl acetate, tocopheryl linoleate, tocopheryl nicotinate, linoleic acid, coenzyme Q-10, resveratrol, a plant extract essential oil, ursolic acid, oleanolic acid, oil-soluble licorice root, and lipoic acid	
7	WO2021040645A1	An anti-aging cosmetic product	An anti-aging chemical compound, showing anti-radical and antioxidant effects, as ethosome formulation	Ethosome nanocarrier formulation for an anti-aging dermo-cosmetic product which shows increasing anti-radical and antioxidant activity, where *Melissa officinalis* L extract is the active substance	Skin
8	CN103251539B	Nanostructured lipid carrier for composite skin lightener and preparation method of composite skin lightener	A compound skin lightener as cosmetics	NLC for a composite skin lightener loaded on the carrier and comprises the 3 active ingredients phenylethyl resorcinol, turmeric extract and pepper seed extract with a lipid material and an emulsifying agent. Further, the enveloping process is simple, controllable and good in repeatability. It also overcomes the active substances that are not soluble in water and easily degrades in presence of light	Skin
9	KR102013804B1	Cosmetic composition containing cubosome with trifolium repense extract	Cosmetics for preventing aging	A cubosome comprising of cosmetic composition including white clover extract and its characterization methods	Skin
10	JP4995423B2	Artificial low-density lipoprotein carrier for transporting substances across	Increases the delivery of hydrophilic substances particularly for crossing BBB	Relates to the use of artificial low-density lipoprotein (LDL) particles that efficiently target substances and deliver them across the BBB	Brain

		the blood–brain barrier			
11	US10596124B2	Lipid nanoparticle compositions and methods as carriers of cannabinoids in standardized precision-metered dosage forms	Increases cannabinoid transport across hydrophobic mucosa by increasing the bioavailability and decreasing the dose of cannabinoids	Phospholipid nanoparticle of cannabinoids formed from phospholipids and simpler lipids in an unfired sequential process that encapsulates an increased concentration of cannabinoids and creates standardized precision-metered dosage forms of cannabinoids, yielding an increase in cannabinoid transport across hydrophobic mucosa	Skin, Brain, mucosal barriers, etc
12	US9737528B2	Liposomes useful for drug delivery to the brain	Liposome formula containing substituted ammonium and/or polyanion and optionally with a needed therapeutic or imaging entity	Methods of formulating the liposome compositions and also provides the methods and kits for the delivery of liposomal compositions to the brain	Brain
13	US10722463B2	Compositions and methods using same drug for delivering agents into a target organ protected by a blood barrier	Delivering a pharmaceutical agent into a target organ protected by a blood barrier	The amount and the liposomes encompassing the active agent are selected and accumulate in cells of the immune system of the subject to generate liposome-loaded immune cells. Then it becomes activated and crosses the blood barrier and an effective amount of the pharmaceutical agent is released from the liposomes in the target organ of the subject	Brain
14	US20150004196A1	Topical drug delivery using phosphatidylcholine	Formulations and methods for transdermal drug delivery comprising a phosphatidylcholine carrier composition having the drug	A non-liposomal multilamellar liquid crystal carrier comprising phosphatidylcholine, a polypeptide for transdermal delivery to dermal vasculature, the phosphatidylcholine comprising a higher concentration of polyenylphosphatidylcholine than the concentration in food-grade soy lecithin	Skin

15	AU2019201792B2	Lipid nanoparticle compositions and methods as carriers of cannabinoids in standardized precision-metered dosage forms	A lipid particle delivery system containing cannabinoids captured in a stable nanoparticle system comprised of lipids, phospholipids and excipients	A standardized precision-metered dosage form of cannabinoids; having increased cannabinoid transport across hydrophobic mucosa (including nasal mucosa), dermal and epidermal barriers with improved bioavailability and decreased dose of cannabinoids and related adverse effects of cannabinoids also	Skin
16	US20120064136A1	Anti-aging and wrinkle treatment methods using nanoemulsion compositions	A nanoemulsion and related methods for treating, preventing, minimizing and/or diminishing signs of skin aging	The nanoemulsion consists of droplets having an average diameter of less than about 3 μm and the droplets contain an oil phase, an aqueous phase comprising at least one surfactant, at least one organic solvent and water	Skin
